# Prevalence of blood parasites in seabirds - a review

**DOI:** 10.1186/1742-9994-8-26

**Published:** 2011-10-31

**Authors:** Petra Quillfeldt, Elena Arriero, Javier Martínez, Juan F Masello, Santiago Merino

**Affiliations:** 1 Max-Planck-Institut für Ornithologie, Vogelwarte Radolfzell, Schlossallee 2, 78315 Radolfzell, Germany; 2 Departamento de Parasitología, Universidad de Alcalá, Alcalá de Henares, Spain; 3 Departamento de Ecología Evolutiva, Museo Nacional de Ciencias Naturales, Consejo Superior de Investigaciones Científicas, Madrid, Spain

## Abstract

**Introduction:**

While blood parasites are common in many birds in the wild, some groups seem to be much less affected. Seabirds, in particular, have often been reported free from blood parasites, even in the presence of potential vectors.

**Results:**

From a literature review of hemosporidian prevalence in seabirds, we collated a dataset of 60 species, in which at least 15 individuals had been examined. These data were included in phylogenetically controlled statistical analyses of hemosporidian prevalence in relation to ecological and life-history parameters. *Haemoproteus *parasites were common in frigatebirds and gulls, while *Hepatozoon *occurred in albatrosses and storm petrels, and *Plasmodium *mainly in penguins. The prevalence of *Haemoproteus *showed a geographical signal, being lower in species with distribution towards polar environments. Interspecific differences in *Plasmodium *prevalence were explained by variables that relate to the exposure to parasites, suggesting that prevalence is higher in burrow nesters with long fledgling periods. Measures of *Plasmodium*, but not *Haemoproteus *prevalences were influenced by the method, with PCR-based data resulting in higher prevalence estimates.

**Conclusions:**

Our analyses suggest that, as in other avian taxa, phylogenetic, ecological and life-history parameters determine the prevalence of hemosporidian parasites in seabirds. We discuss how these relationships should be further explored in future studies.

## Introduction

Birds are infected by a number of intracellular blood parasites, including Haemosporidia of the genera *Plasmodium*, *Haemoproteus *and *Leucocytozoon*, Haemogregarinidae of the genus *Hepatozoon *and Piroplasmida of the genus *Babesia*. These blood parasites can exert important selection pressure on their hosts through effects on survival [1-3], on reproductive success [e.g., [4-8]], on plumage colouration [e.g., [9,10]], with important ecological and evolutionary consequences, such as changes in community structure [e.g., [11]].

The rate of infection varies greatly among different bird orders [e.g., [12,13]], but the reasons for the wide taxonomic variation in parasite prevalence or diversity are still poorly understood [14,15]. While some avian taxa are heavily affected, apparent absence or scarcity of blood parasites has been reported from others [see [16,17]], especially in avian groups such as seabirds [e.g., [18-22]], swifts [23], waders [24] and parrots [25].

In addition to the apparent phylogenetic bias in the incidence of parasitic infections among bird taxa, there is also some evidence that blood parasites are less common in certain habitats such as the Arctic tundra [e.g., [26]], arid environments [e.g., [27,28]], island environments [e.g.,[29]] or marine environments [e.g., [30-32]]. Several hypotheses have been proposed to explain this absence [16], such as the absence or scarcity of proper vectors, a highly specific association between host and parasites with host switching being infrequent (host-parasite assemblage), host immunological capabilities preventing infection by parasites, and competitive exclusion of blood parasite vectors mediated by ectoparasites.

A number of comparative studies have analysed patterns of blood parasite prevalence across bird taxa, with the aim to understand how the selection pressure from parasitism is linked to ecological and evolutionary traits of their hosts [e.g. [3,14,15,33]]. In the present study, we review information on blood parasites in seabirds. Using phylogenetically controlled statistical analyses we tested if blood parasite prevalence in seabirds is related to the following factors: 1) historical/phylogenetic factors, 2) life history parameters, and 3) ecological parameters.

## Results

### Literature review

From the 113 seabird species listed in Table 1, parasitic infections by hematozoa were found in 31 species (27%). This was similar to the percentage of infected species reported in 60 species with at least 15 individuals sampled (20 species or 33%, *χ^2 ^*= 0.18 *d.f*. 1, *P *= 0.669). The prevalence of multiple infections was very low, as only five host species were reported infected by more than one parasite genus (Additional file 1: Table S1): Fjordland crested penguin *Eudyptes pachyrhynchus *(2), little penguin *Eudyptula minor *(2), African penguin *Spheniscus demersus *(3), magnificent frigatebird *Fregata magnificens *(2) and yellow-legged gull *Larus cachinnans *(2). The other 25 species in which infections were found only had a single kind of parasite. The proportion of species infected differed between bird families (Table 1), from complete absence in some groups such as cormorants (7 species studied), skuas (5 species) and auks (3 species) to 100% of species and populations infected in frigatebirds (4 species studied so far). The average prevalence of haematozoa across all studies here reported (*N *= 231 studies, or 6,656 adults and 1,143 chicks, see Additional file 1: Table S1) was 8.5% (blood smears, PCR and ELISA combined) or 5.7% (based on blood smears only).

**Table 1:** Seabird families, sorted by increasing parasite prevalence

Family (no. species)	species studied	species infected	Mean prevalence (no. studies)	*Plasmodium*	*Haemoproteus*	*Leucozytozoon*	*Hepatozoon*	*Babesia*
Pelecanoididae (4)	1 (25%)	0	0 (N = 1)	-	-	-	-	-
Phaethontidae (3)	3 (100%)	0	0 (N = 7)	-	-	-	-	-
Pelecanidae (8)	1 (13%)	0	0 (N = 2)	-	-	-	-	-
Stercorariidae (7)	5 (71%)	0	0 (N = 8)	-	-	-	-	-
Alcidae (22)	3 (14%)	0	0 (N = 4)	-	-	-	-	-
Procellariidae (72)	16 (22%)	2 (12%)	0.1% (N = 28)	*P. sp*. (2)	-	-	-	-
Phalacrocoracidae (32)	8 (25%)	1 (12%)	1.3% (N = 13)	-	-	*L. vanden-brandeni *(1)	-	-
Hydrobatidae (20)	4 (20%)	1 (25%)	3.3% (N = 6)	-	-	-	*H. sp*. (1)	-
Spheniscidae (19)	19 (100%)	6 (32%)	14.4% (N = 64)	*P. relictum *(5), *P. sp*. (1)	-	*L. tawaki *(2)	-	*B. peircei *(2)
Sulidae (10)	6 (60%)	2 (33%)	7.1% (N = 12)	-	*H. sp*. (1)	-	-	*B. poelea *(2), *B. sp*. (1)
Diomedeidae (14)	7 (50%)	4 (57%)	7.8% (N = 14)	*-*	-	*-*	*H. albatrossi *(4)	*-*
Lariidae (92)	36 (18%)	7 (59%)	9.2% (N = 59)	*P. sp*. (1)	*H. larae *(5), *H. passeris *(1)*, H. sp*. (4)	-	-	*B. bennetti *(1)
Fregatidae (5)	4 (80%)	4 (100%)	27.7% (N = 7)	-	*H. iwa *(3), *H. sp*. (1)	-	-	-
All seabirds (453)	113 (25%)	31 (27%)	8.4% (N = 224)	≥ 1 spp.	≥ 3 spp.	1 spp.	≥ 1 spp.	≥ 3 spp.

Species infected as percentage of species studied. No data were found for sheathbills Chionididae (2 species) and skimmers Rynchopidae (3 species). For details and sources, see Table S1 in Additional file 1.

The occurrence of haemoparasites increased from polar to tropical seabirds (*χ^2 ^*= 10.6, *d.f*. = 3, *P *= 0.031). Blood parasites were absent from Antarctic and arctic seabirds (*N *= 15 species). In the Sub-Antarctic islands, 5 of 21 seabird species (24%) studied were found to have haemoparasites, while 18 of 57 temperate seabird species (32%) and 11 of 25 tropical seabirds (44%) had at least one record (see Additional file 1).

### Phylogenetically controlled analysis

**Figure 1:**
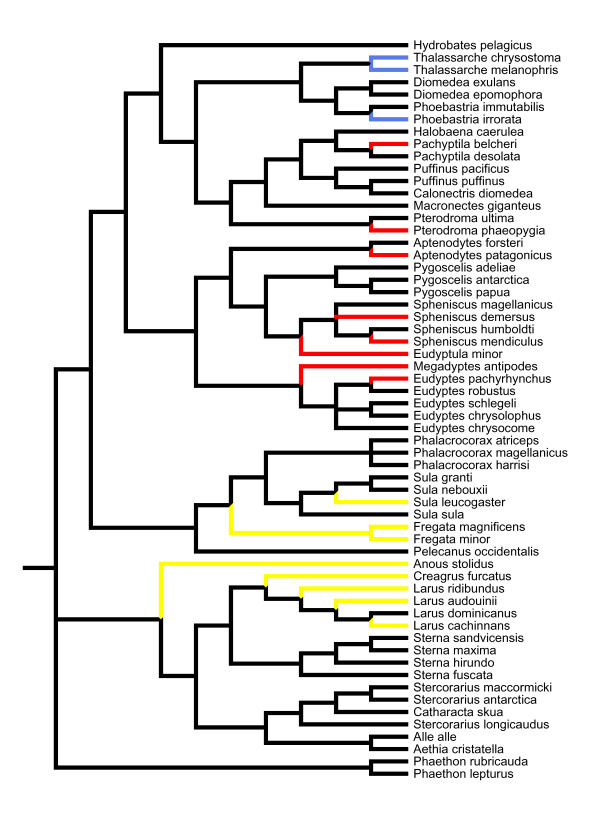
**Phylogeny of seabird species and hemoparasite infections**. The clasification includes species for which at least 15 individuals have been sampled for blood parasites, using blood smears or molecular methods. Colour marked are those species which were found to be infected with *Hepatozoon *(blue), *Plasmodium *(red) and *Haemoproteus *(yellow). The apparent phylogenetic clustering was, however, not statistically significant.

Figure 1 shows phylogenetic associations among species in the study and the information on the presence of parasites for those species. Even though visual inspection suggests phylogenetic clustering of the incidence of parasitism among the different taxa of seabirds (Figure 1), the results of the phylogenetic autoregression analyses did not support this, as interspecific variation in parasite prevalence of the main genera of haematozoa was not explained by phylogenetic associations among species (*Haemoproteus*: rho = -0.13, *P *= 0.292; *Plasmodium*: rho = -0.21, *P *= 0.649; *Hepatozoon*: rho = -1.52, *P *= 0.729). Due to the low prevalence, it was not possible to include *Babesia *in this analysis.

We included the two most commonly observed parasites (*Plasmodium*, *Haemoproteus*) into phylogenetically controlled analyses of hemosporidian prevalence in relation to ecological and life-history parameters. Given their low prevalence, it was not possible to include blood parasites from genera *Hepatozoon *and *Babesia *in this analysis.

Inter-specific differences in *Plasmodium *infections were explained by variables that relate to the exposure to parasites, suggesting that burrow nesters and species with longer nestling periods were more likely to harbour *Plasmodium *(Table 2), and the *Plasmodium *prevalence was also higher in burrow nesters. Method had a significant influence on the proportion of infected individuals detected, with higher prevalences for PCR-based data (Table 2).

**Table 2:** Parameter estimates of phylogenetically informed GLS models and GEE models for *Plasmodium*

Variables in best-fit model	Prevalence (GLS) N = 60 spp. (PCR data included, method included in model)	Presence/absence (GEE) N = 60 spp. (PCR data included)
*Ecological parameters*		
Nesting (burrow = 1, others = 0)	**0.063 ± 0.026, P = 0.018**	**3.408 ± 0.977, P = 0.004**
Foraging (0 = nearshore, 2 = offshore)	- 0.014 ± 0.014, P = 0.301	
*Life-history parameters*		
Mean fledging period (days)	0.042 ± 0.060, P = 0.490	**5.949 ± 2.045, P = 0.011**
*Method *(*smears or PCR*)	**0.051 ± 0.018, P = 0.006**	

Means ± s.e. are given. Only variables retained in the best-fit models are presented in the table. Excluded variables: body mass, chick development, maximum clutch size, mean incubation period, distribution

The presence/absence of infections by *Haemoproteus *was not associated with any of the ecological or life history parameters considered in the study. However, a latitudinal trend was observed in the proportion of individuals infected by *Haemoproteus *(i.e. prevalence), with higher number of individuals infected in species with tropical distribution (Table 3). Method did not influence measured *Haemoproteus *prevalences (Table 3).

**Table 3:** Parameter estimates of phylogenetically informed GLS models and GEE models for *Haemoproteus*

Variables in best-fit model	Prevalence (GLS) N = 60 spp. (PCR data included, method included in model)	Presence/absence (GEE) N = 60 spp. (PCR data included)
*Ecological parameters*		
Distribution (1 = polar, 9 = tropical)	**0.023 ± 0.010, P = 0.025**	0.287 ± 0.209, P = 0.191
*Life-history parameters*		
Mean fledging period (days)	-0.128 ± 0.155, P = 0.413	-1.389 ± 2.00, P = 0.499
*Method *(*smears or PCR*)	-0.013 ± 0.049, P = 0.786	

Means ± s.e. are given. Only variables retained in the best-fit models are presented in the table. Excluded variables: Body mass, Nest site, Foraging, chick development, Maximum clutch size, Mean incubation period

## Discussion

In the present study, we summarize the available information on the prevalence of haematozoa of the genera *Plasmodium*, *Haemoproteus*, *Leucocytozoon*, *Hepatozoon *and *Babesia *in seabirds. As previously suggested [e.g., [18-21]], our review underlines that in general, the incidence of blood parasitic infections is low in seabirds (Table 1 Additional file 1). The average prevalence of haematozoa across all seabird species studied (Additional file 1: Table S1) was 8.7%, compared to 26% in a sample of 14,812 European passerines [14].

### Blood parasites and climate

Blood parasites were absent from all Antarctic and arctic seabirds, and the occurrence increased in milder climates. This finding is in line with previous studies [34] that noted that blood parasites present in sub-Antarctic islands were absent in Antarctica and suggested that this mirrors the absence of suitable vectors in Antarctica [19]. Latitudinal gradients in the prevalence of blood parasites have also been found in other bird species [e.g. [35]], and even in within-species patterns. For example, shorebirds migrating through Europe were free from infections while their conspecifics did show infections in tropical Africa [36].

Haematozoa are transmitted to their vertebrate host through arthropod vectors. Although still a great deal of data about the biology and ecology of various vector species is missing, some authors have suggested that the limit of the distribution of vectors such as mosquitos and sandflies corresponds to the 10°C annual isotherm [e.g., [37]]. Furthermore, for species that hibernate at the larval stage, the -1°C winter isotherm is decisive for their distribution since larvae that freeze do not survive. Thus, vectors would be absent at high latitudes, and more common with higher temperatures, and this is reflected in the distribution of blood parasites in the seabird hosts. Within genera, the present analysis suggested that the prevalence, i.e. the percentage of individuals infected with *Haemoproteus*, was higher in species in more tropical environments, but this was not the case for *Plasmodium*. Given the relatively low detection probability of *Plasmodium *based on blood smears, it is possible that a geographical pattern is not apparent unless more PCR based studies are undertaken. But differences in the biology of the vectors might also explain this difference in the distributon of *Haemoproteus *and *Plasmodium*. While *Plasmodium *is transferred by mosquitoes (Culicidae) and, at least in reptiles, also by sandflies (Psychodidae), *Haemoproteus *is transferred by louse flies (Hippoboscidae) and biting midges (Ceratopogonidae). Some studies have shown an important effect of temperature on activity and host location by these insects [see [38,39]]. However, the identity of vectors is unknown in most avian and, to our knowledge, all seabird studies, and we can expect better insight into parasite distribution from studies of arthropod vectors. For example, ticks (Ixodidae) were generally thought to be the vector for *Hepatozoon*, but one study indicated that fleas can also serve as a vector [40].

### Blood parasites and seabird phylogeny

The most common parasites found in seabirds were *Haemoproteus *and *Plasmodium *(Additional file 1: Table S1), similar to passeriformes [14]. *Hepatozoon *occurred in albatrosses and storm petrels, and *Plasmodium *mainly in penguins, while *Haemoproteus *were especially common in frigatebirds and gulls (Table 1). In this context, it may be relevant that many gulls are adapted to exploiting inland and human-modified or urban environments. These environments may harbour a higher vector density than saline environments [31]. However, the apparent difference among seabird families, though not statistically significant, also suggests a role of immunocompetence in preventing *Haemoproteus *infection in marine birds [16]. This possibility should be analyzed again when data of more seabird species become available. *Babesia *was found in different unrelated seabird species, suggesting the possibility that these infections result from several independent colonization events [e.g.[41]].

### Blood parasites and life-history parameters

In line with the finding that avian *Plasmodium *and their vectors are distributed worldwide except in extreme habitats [12], the occurrence and prevalence of *Plasmodium *infections was independent from the host distribution. However, inter-specific differences were explained by life-history variables that relate to the exposure to parasites, with *Plasmodium *occurrence and prevalence being higher in burrow nesters and occurrence also in seabirds with long nestling periods (Table 3).

Studies in other avian taxa have also suggested that exposure time to vectors is the main factor explaining differences in malaria prevalence: Similar to the present results, parasite species richness in 263 bird species from the Western Palearctic was positively associated with the duration of the nestling period [15]. Moreover, the *Plasmodium *prevalence was associated with the duration of the nestling period [15]. All these data may be indicative of the need of a long exposure time to vectors in nests to allow infection by *Plasmodium *and/or that infections by this parasite occur mainly during nestling stage or nest attending activities.

Moreover, adult shorebirds also showed higher malaria prevalence, suggesting that infection probability increases with cumulative exposure [36]. This is also partially supported by the non-significant tendency for infections being more frequent in birds foraging near-shore, as those expending more time in the vicinity of land are probably more exposed to vectors. Habitat features related to vector availability are also important, and have been used to explain higher or lower blood parasite prevalences in species breeding in forested habitats [33,35,42].

We here found breeding habitat type (burrow) and exposure time at the nest (fledging period) explaining variation in *Plasmodium *but not *Haemoproteus *infections. This might reflect the habitat needs for the different vectors such as mosquitoes (Culicidae) and sandflies (Psychodidae) for *Plasmodium *and louse flies (Hippoboscidae) and biting midges (Ceratopogonidae) in *Haemoproteus*. For example, sandfly larvae often inhabit damp places containing organic matter such as cracks in walls or rock and animal burrows, where they feed on dead organic matter. Adults are blood suckers, but to our knowledge, the actual vectors for *Plasmodium *infections in seabirds are not known, and other arthropod larvae might have similar habitat requirements. The present analysis therefore strongly suggests that the vectors of seabird blood parasites deserve further study if we are to understand distribution patterns.

Previous phylogenetically controlled comparative studies have further identified body mass [14,15,43] and embryonic development period [15,33] as explanatory variables for parasite prevalence. The latter might be due to enhanced immune performance in more slowly developing birds with longer embryonic development periods [44]. Although no such relationship was found in the present study, the overall low prevalence across the generally long-lived, slowly developing seabird taxa strongly suggests that such a mechanism also works in seabirds. It should also be noted that the relationship was not found for any parasite genus analysed separately in Western Paleartic birds [15].

Slightly different variables enter in the best-fit models predicting interspecific variation in parasite impact in seabirds, when the dependent variable is parasite prevalence (continuous variable) or the presence/absence of infections (categorical variable). These differences might be due to the fact that the analyses with the variable presence/absence ignore variation among infected species (as they are all scored as 1), and also stress the need for more data to obtain more robust patterns across species. Alternatively, the differences might be explained by traits concerning the life cycle of blood parasites or their vectors, which deserve more study.

## Conclusions

In summary, seabirds are long-lived with a relatively slow-life history and low rates of haemoparasite infection. Partly owing to the scarcity of infection, no comparative information on parasite prevalences had been reported so far for most seabird taxa. The present results show that multiple factors are responsible for patterns of association between parasitic infections and ecological and life history traits in seabirds. Life history parameters and ecological parameters that show some correlation with parasite abundance seem to be associated to the abundance and/or life cycle requirements of specific vectors. The findings on nesting habitat and exposure time, in particular, are well in line with studies across avian taxa. To better understand the underlying ecological relationships, however, efforts are now needed to identify the arthropod vectors and gain a better knowledge of their distribution and biology.

Historical/phylogenetic factors also play a role, as indicated by the high prevalence of *Haemoproteus *in gulls and frigatebirds, the apparent confinement of *Hepatozoon *to albatross and storm-petrel species, and the high prevalence of *Plasmodium *in penguins. Studies of the phylogentic relationships of these parasites will be instructive in order to understand the evolution of such host-parasite associations.

## Methods

### Literature review

In first reviews on infections by the main genera of haemoparasites in different bird species [45,46] as well as later updates [e.g., [13,47]] no data on prevalence or sample sizes were given, and many data from birds in captivity were included. In our present review (Additional file 1), we included only data of studies on wild-caught birds. We checked all the original references for this point, and references of birds of uncertain origin (e.g. bird rescue stations) were not included. Prevalence of current or past infections is measured as the proportion of individuals infected with haematozoa, recorded either by microscopic inspection of blood smears or by molecular methods that detect antibodies (ELISA) or genetic material of the haematozoa (PCR).

In the last decade, numerous studies have focused in molecular detection of blood parasitic infections in birds, mainly by amplifying DNA of the parasite by PCR [e.g., [22,48-50]], or by immunological detection of the presence of specific antibodies by ELISA [e.g., [51]]. Although these techniques are more sensitive, especially in detecting low intensity infections that may have passed unnoticed by microscopy [e.g., [52]], the small number of seabird species with molecular data available to date, does not allow comparative analyses at a broad taxonomic scale. However, we reviewed the existing literature in molecular detection of blood parasites in seabirds. There were only two cases (penguin species) that we classified as having zero prevalence of blood parasites, but that have been reported as positive using molecular techniques (Figure 1).

### Phylogenetically controlled analysis

#### a) Variables and database

In the phylogenetically controlled comparative models, we included life history and ecological variables that could explain inter-specific differences in prevalence of avian hematozoa. The variables we used were extracted from those available in reviews on bird body masses [53] and seabird biology [54]: (1) average body mass, (2) ecological parameters: (a) distribution on a scale from 1 = polar to 9 = tropical, (b) nesting habits, as either "1" for burrow or crevice nesters, or "0" for open nesters, including open ground, cliff, marsh or tree nesters, (c) foraging distribution on a scale from 0 = nearshore to 2 = offshore, and (3) Life-history parameters: (a) chick developmental mode on a scale from 0 = altricial, 1 = semialtricial, 2 = semiprecocial and 3 = precocial, (b) maximum clutch size, (c) incubation period (days) and (d) mean fledging period (days).

To control for the effect of sample size on the reliability of estimates of parasite prevalence, we restricted our comparative analyses to species for which at least 15 individuals have been sampled for blood parasites, using either blood smears [55], PCR, or both methods.

Three species (Silver gulls *Larus novaehollandiae*, Dolphin gulls *Larus scoresbii *and Little Pied cormorants *Phalacrocorax melonoleucos*) were excluded from the analyses, as we could not find information on all life history parameters for the analyses for these species.

The timing of blood sampling can affect the observed blood parasite prevalence in many species. Several weeks or months after infection, blood parasites can enter a latent stage, when the parasites disappear from the peripheral blood, but can persist in the internal organs. Relapses are usually synchronized with the breeding period of birds [13]. As most seabirds can only be accessed during the breeding season, the degree of parasitemia is then expected to be highest.

#### b) Phylogeny

We constructed a phylogenetic tree using published information on phylogenetic associations among the species of our study, in particular for Neoaves [56,57], for Sphenisciformes [58], for Procellariiformes [59-63], for Phaethontidae [64], for Pelecaniformes [65-68] and for Charadriiformes [69-77].

Because we used information from different sources and the branch lengths were not specified in all studies we used to reconstruct the phylogeny, we conducted all analyses with a tree of equal branch lengths, which assumes a punctuational model of evolution [78]. The software MESQUITE [79] was used to construct the phylogenetic tree (see tree topology in Figure 1).

#### c) Statistical analyses

We included all available data (from blood smears and PCR data) for each species with at least 15 sampled individuals. Parasite prevalences were arsin-square root transformed, while the variables body mass, clutch size, incubation and nestling period were log_10_-transformed. To control for allometric effects on parasite prevalence, average adult body mass for the species (obtained from the literature) was included in all analyses as a predictor variable. Evolutionary models were tested by Generalized Least Squares models fitted by REML, incorporating phylogenetic information with a Brownian motion correlation structure, and method (smears vs. PCR) as a variable. Model selection was based on Akaike's information criterion (AIC). All analyses were repeated using parasite prevalence as a categorical variable (presence/absence) in phylogenetically informed Generalized Estimating Equations (GEE) models [80]. To estimate parameters of character evolution and the proportion of variance in parasite prevalence explained by phylogenetic associations among species, we computed phylogenetic autorregressions and calculated Moran's autocorrelation Index [81]. We used R 2.11.1 (R Development Core Team 2010) and the package "ape" [82] for the analyses.

## Competing interests

The authors declare that they have no competing interests.

## Authors' contributions

PQ, JFM, JM and SM conceived and designed the study. PQ assembled the database from the literature. EA carried out the bioinformatic analyses. PQ and EA were responsible for data analysis and drafted the manuscript. JFM, JM and SM contributed to the final draft of the manuscript. All authors read and approved the final manuscript.

## Supplementary Material

Additional file 1 **Table S1. Studies of intracellular hematozoa in wild seabirds**. Most studies were conducted by examination of blood smears. Any other techniques (ELISA, PCR) are detailed in the column "method" together with the target Genus (P-*Plasmodium*, L-*Leucocytozoon*, H-*Hemoproteus*). Prevalence was added in brackets where known [83-143]. Click here for file
