# Supplementary material for: Prevalence of blood parasites in seabirds - a review
Source: Front Zool. 2011 Oct 31;8:26. doi: 10.1186/1742-9994-8-26 (PMC3223496; doi:10.1186/1742-9994-8-26)
Supplement: Additional file 1 — Table S1. Studies of intracellular hematozoa in wild seabirds. Most studies were conducted by examination of blood smears. Any other techniques (ELISA, PCR) are detailed in the column "method" together with the target Genus (P-Plasmodium, L-Leucocytozoon, H-Hemoproteus). Prevalence was added in brackets where known [83-143]. [file 1742-9994-8-26-S1.PDF]

Electronic Supplementary Material

Table S1. Studies of intracellular hematozoa in wild seabirds. Most studies were conducted by examination of blood smears. Any other techniques (ELISA, PCR) are detaileded in the column “method” together with the and target Genus (P-*Plasmodium*, L-*Leucocytozoon*, H-*Heamoproteus*). Prevalence noted in brackets where known. See footnote (page 22) for abbreviations.

Species		Sampling site	Region	N (ad)	N (chicks)	Parasites found	Method	Ref Nr.
**<u>Penguins Spheniscidae</u>**								
Emperor Penguin	*Aptenodytes forsteri*	McMurdo Sound	AA	1		NF		83
		near Mawson Station	AA	50		NF		84
King Penguin	*Aptenodytes patagonicus*	Macquarie I.	SAA	34		NF		85
		Heard I.	SAA	8		NF		85
		South Georgia	SAA	44		NF		86
		Macquarie I.	SAA	17		NF		87

Species		Sampling site	Region	N (ad)	N (chicks)	Parasites found	Method	Ref Nr.
Southern Rockhopper Penguin	*Eudyptes chrysocome*	Isla Pinguino	TEMP	40		NF		88
		Macquarie I.	SAA	42		NF		87
		Campbell I.,	SAA	9		NF	ELISA (P)	89
		New I., Falkland Is.	SAA	28		NF	PCR (P,L,H)	22
Eastern Rockhopper Penguin	*Eudyptes chrysocome filholi*	Marion I.	TEMP	80		NF		90
Northern Rockhopper Penguin	*Eudyptes moseleyi*	Gough I.	TEMP	5		*Plasmodium relictum (20%)*		91
Macaroni Penguin	*Eudyptes chrysolophus*	Heard I.	SAA	12		NF		85
		Marion I.	TEMP	89		NF		90
Fjordland Crested Penguin	*Eudyptes pachyrhynchus*	South lsland, NZ	TEMP	18		*Leucocytozoon tawaki (94%)*		92
		Snares I., NZ	SAA	28		*Plasmodium relictum (11%)*		93
Snares Island Penguin	*Eudyptes robustus*	Snares I., NZ	SAA	25		NF		92
Royal Penguin	*Eudyptes schlegeli*	Macquarie I.	SAA	28		NF		85
		Macquarie I.	SAA	40		NF		87

Species		Sampling site	Region	N (ad)	N (chicks)	Parasites found	Method	Ref Nr.
Erect-crested Penguin	*Eudyptes sclateri*	Snares I., NZ	SAA	1		NF		92
Little Penguin	*Eudyptula minor*	Southern Tasmania	TEMP	54	3	NF		94
		Southern Australia	TEMP	NG		*Babesia peircei*		95
		Trio I., NZ	TEMP	1		NF		93
		New South Wales	TEMP	3	15	NF		96
		Low Head, Tasmania	TEMP	70		NF		84
		Penguin I., W Australia	TEMP	30		NF		84
		Codfish I., NZ	TEMP	8		*Plasmodium sp.* (63%)	ELISA (P)	89
Yellow-eyed Penguin	*Megadyptes antipodes*	Foveaux Strait, NZ	TEMP	2		*Plasmodium relictum* (50%)		93
		Otago Peninsula, NZ	TEMP	143		NF		97
		Foveaux Strait, NZ	TEMP	10		*Plasmodium relictum* (10%)		91
		Codfish I., NZ	TEMP	32		*Plasmodium sp.* (91%)	ELISA (P)	89
		Catlins, NZ	TEMP	24		*Plasmodium sp.* (79%)	ELISA (P)	89

Species		Sampling site	Region	N (ad)	N (chicks)	Parasites found	Method	Ref Nr.
Yellow-eyed Penguin		Campbell I.,	SAA	22		*Plasmodium sp.* (23%)	ELISA (P)	89
		Mainland South I., NZ	TEMP	20		*Plasmodium sp.* (55%)	ELISA (P)	89
		Enderby I., NZ	TEMP	20		*Plasmodium sp.* (65%)	ELISA (P)	89
		Banks Peninsula, NZ	TEMP	16		*Plasmodium sp.* (81%)	ELISA (P)	89
		Otago Peninsula, NZ	TEMP	11		*Plasmodium sp.* (82%)	ELISA (P)	89
		Mainland South I., NZ	TEMP	17	18	NF		50
		Mainland South I., NZ	TEMP	95		NF	PCR (L)	50
		Stewart Island, NZ	TEMP	12	2	NF		50
		Stewart Island, NZ	TEMP	12	7	*Leucocytozoon sp.* (89%)	PCR (L)	50
Adelie Penguin	*Pygoscelis adeliae*	McMurdo Sound	AA	18		NF		83
		Cape Royds	AA	94		NF		98
		Bechervaise I.	AA	26		NF		84

Species		Sampling site	Region	N (ad)	N (chicks)	Parasites found	Method	Ref Nr.
Chinstrap Penguin	*Pygoscelis antarctica*	South Georgia	SAA	1		NF		86
		Deception I.	AA	40	58	NF		19
Gentoo Penguin	*Pygoscelis papua*	Macquarie I.	SAA	74		NF		85
		South Georgia	SAA	50		NF		86
		Macquarie I.	SAA	22		NF		87
		New I., Falkland Is.	SAA	15		NF	PCR (P,L,H)	88
African penguin	*Spheniscus demersus*	Near Cape Town, SA	TEMP	34		*Plasmodium relictum* (6%)		91
		Marcus I., Cape Prov., SA	TEMP	21		NF		99
		Dyer I., Cape Prov., SA	TEMP	42		NF		99
		Robben Island, SA	TEMP	46		*Plasmodium sp.* (35%)	ELISA (P)	51
		Dassen Island, SA	TEMP	30		*Plasmodium sp.* (33%)	ELISA (P)	51
		Boulders, Simons Town, SA	TEMP	34		*Plasmodium sp.* (29%)	ELISA (P)	51

Species		Sampling site	Region	N (ad)	N (chicks)	Parasites found	Method	Ref Nr.
Humboldt Penguin	*Spheniscus humboldti*	Algarrobo, Chile	TEMP	30		NF		100
		Cachagua I., Chile	TEMP	21		NF		100
		Cachagua I., Chile	TEMP	18		NF		84
Magellanic Penguin	*Spheniscus magellanicus*	Chubut, Argentina	TEMP	36	300	NF		32
		New I., Falkland Is.	SAA	18		NF	PCR (P,L,H)	22
Galapagos Penguin	*Spheniscus mendiculus*	Galápagos	TROP	94		NF	PCR (P)	101
		Galápagos	TROP	330		NF		102
		Galápagos	TROP	401		*Plasmodium* (5%)	PCR (P)	103
**<u>Albatrosses Diomedeidae</u>**								
Buller's Albatross	*Diomedea bulleri*	Wellington, NZ	TEMP	1		NF		93
Grey-headed Albatross	*Diomedea chrysostoma*	South Georgia	SAA	100		*Hepatozoon albatrossi* (1%)		86
Royal Albatross	*Diomedea epomophora*	Wellington, NZ	TEMP	2		NF		93
		Otago Peninsula, NZ	TEMP	14		NF		97
		Campbell Island, NZ	TEMP	19		*Hepatozoon albatrossi* (79%)		104

Species		Sampling site	Region	N (ad)	N (chicks)	Parasites found	Method	Ref Nr.
Wandering Albatross	*Diomedea exulans*	Ninety-mile beach, NZ	TEMP	2		NF		93
		South Georgia	SAA	50		*Hepatozoon albatrossi* (26%)		86
Laysan Albatross	*Diomedea immutabilis*	Tern I., French Frigate Shoals	TROP	16		NF		105
		Midway atoll	TROP	43	41	NF		105
		Kauai	TROP		31	NF		105
		Laysan I., Hawaii	TROP	20		NF		105
Waved Albatross	*Diomedea irrorata*	Española, Galápagos	TROP	45		NF		106
Black-browed Albatross	*Diomedea melanophris*	Wellington, NZ	TEMP	1		NF		93
		South Georgia	SAA	90		*Hepatozoon albatrossi* (3%)		86
**Petrels and shearwaters Procellariidae**								
Cory's Shearwater	*Calonectris diomedea*	Aegean	TEMP	8		NF		107
		Chafarinas Is.	TEMP	38		NF		108
		Berlenga I.	TEMP		15	NF	PCR (P,L,H)	22

Species		Sampling site	Region	N (ad)	N (chicks)	Parasites found	Method	Ref Nr.
Blue Petrel	*Halobaena caerulea*	South Georgia	SAA	48		NF		86
Southern Giant Petrel	*Macronectes giganteus*	Chubut, Argentina	TEMP	23		NF		32
		Wellington, NZ	TEMP	1		NF		93
		South Georgia	SAA	7		NF		86
Northern Giant Petrel	*Macronectes halli*	South Georgia	SAA	7		NF		86
Thin-billed Prion	*Pachyptila belcheri*	New I., Falkland Is.	SAA	28		*Plasmodium sp.* (4%)	PCR (P,L,H)	22
Dove Prion	*Pachyptila desolata*	South Georgia	SAA	52		NF		86
		Wellington, NZ	TEMP	2		NF		93
Fairy Prion	*Pachyptila turtur*	Wellington, NZ	TEMP	2		NF		93
Snow Petrel	*Pagodroma nivea*	McMurdo Sound	AA	11		NF		83
White-chinned Petrel	*Procellaria aequinoctialis*	South Georgia	SAA	3		NF		86
Trinidade Petrel	*Pterodroma arminjoniana*	Ducie, Pitcairn Is.	TROP	3	9	NF		18
		Mauritius	TROP	1		NF		109
Kermadec Petrel	*Pterodroma neglecta*	Ducie, Pitcairn Is.	TROP	2	10	NF		18

Species		Sampling site	Region	N (ad)	N (chicks)	Parasites found	Method	Ref Nr.
Hawaiian Petrel	*Pterodroma phaeopygia*	Kauai, Hawaii	TROP	1		*Plasmodium sp.*		110
		Maui, Hawaii	TROP	22		NF		111
		Maui , Hawaii	TROP	28	46	NF		105
Murphy's Petrel	*Pterodroma ultima*	Ducie, Pitcairn Is.	TROP		10	NF		18
		Oeno, Pitcairn Is.	TROP	20		NF		18
Sooty Shearwater	*Puffinus griseus*	Trio I., NZ	TEMP	7		NF		86
		offshore Is. of northern Mexico	TEMP	1		NF		112
Wedge-tailed Shearwater	*Puffinus pacificus*	Oahu, Hawaii	TROP	45	45	NF		105
Manx Shearwater	*Puffinus puffinus*	Western Europe	TEMP	401		NF		113
		Skomer I.	TEMP	12		NF	PCR (P,L,H)	22
		offshore Is. of northern Mexico	TEMP	2		NF		112

Species		Sampling site	Region	N (ad)	N (chicks)	Parasites found	Method	Ref Nr.
**<u>Storm-petrels Hydrobatidae</u>**								
Black-bellied Storm Petrel	*Fregetta tropica*	South Georgia	SAA	1		NF		86
British Storm Petrel	*Hydrobates pelagicus*	Mitjana I. and Tabarca I.	TEMP		25	NF		114
		Benidorm	TEMP	55	63	NF		20
Wilson's Storm Petrel	*Oceanites oceanicus*	McMurdo Sound	AA	1		NF		83
		King George I.	AA	12		NF	PCR (P,L,H)	22
Black Storm Petrel	*Oceanodroma melania*	offshore Is. of northern Mexico	TEMP	5		*Hepatozoon sp. (20%)*		112
**<u>Diving petrels Pelecanoididae</u>**								
Common Diving Petrel	*Pelecanoides urinatrix*	Wellington, NZ	TEMP	2		NF		93
**<u>Tropicbirds Phaethontidae</u>**								
White-tailed Tropicbird	*Phaethon lepturus*	Aldabra Atoll, Indian Ocean	TROP	15		NF		115

Species		Sampling site	Region	N (ad)	N (chicks)	Parasites found	Method	Ref Nr.
Red-tailed Tropicbird	*Phaethon rubricauda*	Johnston Atoll	TROP	36	65	NF		105
		Tern I., French Frigate Shoals	TROP	53		NF		105
		Aldabra Atoll, Indian Ocean	TROP	8		NF		115
		Oeno, Pitcairn Is.	TROP		20	NF		18
		Christmas I.	TROP	12		NF	PCR (P,L,H)	22
Red-billed Tropicbird	*Phaethon aethereus*	offshore Is. of northern Mexico	TEMP	1		NF		112
<u>**Pelicans Pelecanidae**</u>								
Brown Pelican	*Pelecanus occidentalis*	east coast of Florida	TEMP	35		NF		116
		offshore Is. of northern Mexico	TEMP	29		NF		112
<u>**Gannets and boobies Sulidae**</u>								
Abbott's Booby	*Papasula abbotti*	Christmas I.	TROP	12		NF	PCR (P,L,H)	22

Species		Sampling site	Region	N (ad)	N (chicks)	Parasites found	Method	Ref Nr.
Masked Booby	*Sula dactylatra*	Desnoeufs, Amirantes, W Indian Ocean	TROP	9		*Babesia sp.* (22%)		117
Brown Booby	*Sula leucogaster*	Johnston Atoll	TROP	70	35	*Babesia poelea* (54% in chicks, 13% in adults)		118
		offshore Is. of northern Mexico	TEMP	1		NF		112
		Christmas I.	TROP	12		NF	PCR (P,L,H)	22
Blue-footed Booby	*Sula nebouxii*	offshore Is. of northern Mexico	TEMP	19		NF		112
Red-footed Booby	*Sula sula*	Oahu, Hawaii	TROP	35	34	NF		105
		Aldabra Atoll, Indian Ocean	TROP	28		NF		115
		Genovesa, Galápagos	TROP	23		*Haemoproteus* (9%)		119
		Oeno, Pitcairn Is.	TROP		15	NF		18
		Christmas I.	TROP	12		NF	PCR (P,L,H)	22

Species		Sampling site	Region	N (ad)	N (chicks)	Parasites found	Method	Ref Nr.
Nazca booby	*Sula granti*	Genovesa, Galápagos	TROP	25		NF		119
**<u>Cormorants Phalacrocoracidae</u>**								
Flightless Cormorant	*Phalacrocorax harrisi*	Galápagos	TROP	448		NF		102
Imperial Shag	*Phalacrocorax atriceps*	Chubut, Argentina	TEMP	36	17	NF		32
		South Georgia	SAA	1		NF		86
		Macquarie I.	SAA	42		NF		87
		New I., Falkland Is.	SAA	25		NF	PCR (P,L,H)	22
Double-crested Cormorant	*Phalacrocorax auritus*	northeastern Mexico	TEMP	1		NF		120
		Baker county, SW Georgia	TEMP	3		NF		121
		offshore Is. of northern Mexico	TEMP	4		NF		112
Guanay Cormorant	*Phalacrocorax bougainvillei*	Chubut, Argentina	TEMP	2	0	NF		32
Great Cormorant	*Phalacrocorax carbo*	Lake Wairarapa, NZ	TEMP	1		NF		93
Rock Cormorant	*Phalacrocorax magellanicus*	Chubut, Argentina	TEMP	16	14	NF		32

Species		Sampling site	Region	N (ad)	N (chicks)	Parasites found	Method	Ref Nr.
Little Pied Cormorant	*Phalacrocorax melanoleucos*	Mildura, Victoria, Australia	TEMP	35	NG (incl.)	*Leucocytozoon vandenbrandeni* (17%)		122
Olivaceous Cormorant	*Phalacrocorax olivaceus*	Chubut, Argentina	TEMP	3	0	NF		32
**<u>Frigatebirds Fregatidae</u>**								
Christmas I. Frigatebird	*Fregata andrewsi*	Christmas I.	TROP	9		*Haemoproteus* (56%)	PCR (P,L,H)	22
Magnificent Frigatebird	*Fregata magnificens*	offshore Is. of northern Mexico	TEMP	15		*Hepatozoon sp.* (7%)		112
		Isla Isabel, Mexico	TROP	251		*Haemoproteus iwa* (16%)		123
Great / Lesser Frigatebird	*Fregata minor / F.ariel*	Aldabra Atoll, Indian Ocean	TROP	NG	62	*Haemoproteus* (15%)		115
Great Frigatebird	*Fregata minor*	Genovesa, Galápagos	TROP	24		*Haemoproteus* (29%)		119
		Tern I., French Frigate Shoals	TROP	20	10	*Haemoproteus iwa* (36%)		124
		Laysan I., Hawaii	TROP	40	20	*Haemoproteus iwa* (35%)		124

Species		Sampling site	Region	N (ad)	N (chicks)	Parasites found	Method	Ref Nr.
**Skuas Stercorariidae**								
Brown Skua	*Stercorarius antarctica*	Chubut, Argentina	TEMP	14	13	NF		32
		South Georgia	SAA	1		NF		86
South Polar Skua	*Stercorarius maccormicki*	Vestfold Hills	AA	125		NF		125
		McMurdo Sound	AA	17		NF		83
Great Skua	*Catharacta skua*	Foula Shetland	TEMP	46		NF		126
		St. Kilda	TEMP	53		NF		126
Long-tailed Skua	*Stercorarius longicaudus*	Prince of Wales I., Canada	A	17		NF		98
Arctic Skua	*Stercorarius parasiticus*	Prince of Wales I., Canada	A	2		NF		98

Species		Sampling site	Region	N (ad)	N (chicks)	Parasites found	Method	Ref Nr.
**<u>Gulls and terns Lariidae</u>**								
Common Noddy	*Anous stolidus*	Aldabra Atoll, Indian Ocean	TROP	24		*Haemoproteus* (4%)		115
		Oeno, Pitcairn Is.	TROP	1		NF		18
		Bird I., Seychelles, W Indian Ocean	TROP	1		NF		117
		offshore Is. of northern Mexico	TEMP	2		NF		112
Black Tern	*Chlidonias niger*	Western Europe	TEMP	NG		NF		127
		Lake Okaboji		2		NF		128
Swallow-tailed Gull	*Creagrus furcatus*	Genovesa, Galápagos	TROP	19		*Haemoproteus* (16%)		119
Dolphin Gull	*Larus scoresbii*	Chubut, Argentina	TEMP	7	10	NF		32
		New I., Falkland Is.	SAA	20		*Haemoproteus* (5%)	PCR (P,L,H)	22
Gull-billed Tern	*Gelochelidon nilotica*	India				NF		129

Species		Sampling site	Region	N (ad)	N (chicks)	Parasites found	Method	Ref Nr.
White Tern	*Gygis alba*	Aldabra Atoll, Indian Ocean	TROP	1		NF		115
			TROP			NF		130
		Oeno, Pitcairn Is.	TROP		1	NF		18
Herring Gull	*Larus argentatus*	Western Europe	TEMP	4		NF		113
Yellow-legged Gull	*Larus cachinnans*	Medes Is.	TEMP	155		*Haemoproteus larae* (89%)		131
		Chafarinas Is.	TEMP	6		*Haemoproteus larae* (100%)		132
		Ebro Delta	TEMP	26		*Haemoproteus larae* (27%)		132
		Benidorm	TEMP	22		*Haemoproteus larae* (100%) and *Babesia bennetti* (5%)		133
		Columbretes I.	TEMP	13		*Haemoproteus larae* (39%)		133
		Benidorm	TEMP		2	*Babesia bennetti*		134
		Benidorm	TEMP		34	*Babesia bennetti* (3%)		113
Audouin's Gull	*Larus audouinii*	Chafarinas Is.	TEMP	46		*Haemoproteus larae* (92%)		132
		Ebro Delta	TEMP	44		*Haemoproteus larae* (29%)		132

Species		Sampling site	Region	N (ad)	N (chicks)	Parasites found	Method	Ref Nr.
Olrog's Gull	*Larus atlanticus*	Chubut, Argentina	TEMP		2	NF		32
Californian Gull	*Larus californicus*	offshore Is. of northern Mexico	TEMP	1		NF		112
Common Gull	*Larus canus*	Western Europe	TEMP	2		NF		113
Black-tailed Gull	*Larus crassirostris*	South Korea	TEMP			*Haemoproteus*	PCR	135
Ring-billed Gull	*Larus delawarensis*	Lake Okaboji, Iowa	TEMP	2		NF		128
Kelp Gull	*Larus dominicanus*	Chubut, Argentina	TEMP	6	23	NF		32
			AA			NF		136
		Lake Wairarapa, NZ	TEMP	20		NF		93
Lesser Black-backed Gull	*Larus fuscus*	Western Europe	TEMP	3		NF		113
Heermann's Gull	*Larus heermanni*	offshore Is. of northern Mexico	TEMP	1		NF		110
Glaucous Gull	*Larus hyperboreus*	Prince of Wales I., Canada	A	6		NF		98
Greater Black-backed Gull	*Larus marinus*	Western Europe	TEMP	NG		NF		127

Species		Sampling site	Region	N (ad)	N (chicks)	Parasites found	Method	Ref Nr.
Little Gull	*Larus minutus*	Iraq	TEMP			NF		137
Silver Gull	*Larus novaehollandiae*	Wellington, NZ	TEMP	20		NF		93
Western Gull	*Larus occidentalis*	offshore Is. of northern Mexico	TEMP	14		NF		112
Bonaparte's Gull	*Larus philadelphia*	offshore Is. of northern Mexico	TEMP	1		NF		112
Black-headed Gull	*Larus ridibundus*	South Turkmenistan	TEMP			*Haemoproteus passeris*		138
		Kazakhstan	TEMP			*Haemoproteus larae*		139
		Western Europe	TEMP	22		*Haemoproteus larae* (5%)		113
		SE Kazakhstan	TEMP	23		*Haemoproteus larae* (9%)		140
Thayer's Gull	*Larus thayeri*	Prince of Wales I., Canada	A	3		NF		98
Black-legged Kittiwake	*Rissa tridactyla*	Western Europe	TEMP	1		NF		113
Little Tern	*Sterna albifrons*	Kazakhstan	TEMP			*Haemoproteus larae*		139
		India				NF		129

Species		Sampling site	Region	N (ad)	N (chicks)	Parasites found	Method	Ref Nr.
Indian River Tern	*Sterna aurantia*	India				NF		129
Forster's Tern	*Sterna forsteri*	Lake Okaboji, Iowa	TEMP	4		*Plasmodium sp.* (25%)		128
Sooty Tern	*Sterna fuscata*	Johnston Atoll	TROP	37	35	NF		105
		Bird I., Seychelles, W Indian Ocean	TROP	3		NF		117
		Desnoeufs, Amirantes, W Indian Ocean	TROP	17		NF		117
		Goelette I., Farquhar Atoll, W Indian Ocean	TROP			NF		117
		Ducie, Pitcairn Is.	TROP		6	NF		18
		offshore Is. of northern Mexico	TEMP	50		NF		112

Species		Sampling site	Region	N (ad)	N (chicks)	Parasites found	Method	Ref Nr.
Common Tern	*Sterna hirundo*	Kazakhstan	TEMP			*Haemoproteus larae*		139
		India				NF		129
		Western Europe	TEMP	NG		NF		127
		Cape Cod, Massachusetts, US	TEMP	75		NF		141
Arctic Tern	*Sterna paradisaea*	Prince of Wales I., Canada	A	5		NF		98
Lesser Crested Tern	*Sterna bengalensis*	India				NF		129
Royal Tern	*Sterna maxima*	Chubut, Argentina	TEMP		21	NF		32
Sandwich Tern	*Sterna sandvicensis*	Chubut, Argentina	TEMP	11	6	NF		32
		Western Europe	TEMP	1		NF		113
Sabine's Gull	*Xema sabini*	Prince of Wales I., Canada	A	8		NF		98
**Auks Alcidae**								
Crested Auklet	*Aethia cristatella*	Talan I.	A	131		NF		142

Species		Sampling site	Region	N (ad)	N (chicks)	Parasites found	Method	Ref Nr.
Little Auk	*Alle alle*	Svalbard	A	100		NF		143
			A	60		NF	PCR (P,L,H)	143
Brünnich's Guillemot	*Uria lomvia*	Western Europe	TEMP	NG		NF		127

NG = not given, NF = not found. Regions: A=Arctic, AA=Antarctic, SAA=Subantarctic, TEMP=Temperate, TROP=Tropical.
